# Synthesis of Electrocatalytic Tungsten Carbide Nanoparticles by High-Pressure and High-Temperature Treatment of Organotungsten Compounds

**DOI:** 10.3390/nano15030170

**Published:** 2025-01-23

**Authors:** Taijiro Tadokoro, Sota Sato, Ichiro Yamane, Hiroki Waizumi, Seiya Yokokura, Toshihiro Shimada

**Affiliations:** 1 Graduate School of Chemical Science and Engineering, Hokkaido University, Kita 13 Nishi 8, Kita-ku, Sapporo 060-8628, Japan; taijiro0209-2001@eis.hokudai.ac.jp (T.T.); hiroki.waizumi@eng.hokudai.ac.jp (H.W.); seiyayokokura@eng.hokudai.ac.jp (S.Y.); 2 Division of Applied Chemistry, Faculty of Engineering, Hokkaido University, Kita 13 Nishi 8, Kita-ku, Sapporo 060-8628, Japan; satou.souta.k2@elms.hokudai.ac.jp

**Keywords:** tungsten carbide, electrocatalysts, ORR, high-pressure high-temperature pyrolysis

## Abstract

Metal–organic framework (MOF)-derived carbon, which contains metal nanoparticles embedded in a carbon matrix, is becoming an important group of catalysts. We report the synthesis of tungsten carbide–carbon nanocomposites using a similar concept, i.e., by pyrolysis of organotungsten compounds under high-temperature and high-pressure conditions. We characterized the product using various analytical techniques and examined its electrocatalytic activity. Two precursors, Bis(cyclopentadienyl)tungsten (IV) dichloride (Cp_2_WCl_2_) and Bis(cyclopentadienyl)tungsten (IV) dihydride (Cp_2_WH_2_) were pyrolyzed at 4.5 GPa and 600 °C. Tungsten carbide (*β*-WC_1−x_) crystals with a size of 2 nm embedded in graphitic carbon were formed from Cp_2_WH_2_-derived samples. Electrochemical measurements showed that all samples were active in the oxygen reduction reaction (ORR), with the Cp_2_WH_2_-derived sample having the best catalytic performance.

## 1. Introduction

With the escalation of climate change and the growing need for clean and sustainable energy production to address it, improving the performance of related catalysts is attracting increasing attention. In particular, precious metals such as Pt, Pd, and Rh have been widely used as energy-related catalysts. These metals exist in limited quantities on the upper crust and are extremely expensive [1,2,3]. This is one of the reasons that hindered the widespread use of the new processes on a large scale.

Among them, there is a growing demand for electrocatalysts for oxygen reduction reactions (ORRs) in fuel cells and metal–air batteries, as well as water-splitting, which play an essential role in clean energy technologies [4,5,6]. There is an urgent need to develop high-performance ORR catalysts using more affordable and abundant materials than precious-metal-based catalysts.

Transition metal carbides, especially tungsten carbides, have received considerable attention as an alternative electrocatalyst, co-catalyst, or support material for precious metals [7,8,9]. They are characterized by high catalytic activity, low cost, and excellent electrochemical corrosion resistance [10,11,12,13]. However, the synthesis of transition metal carbides generally requires high temperatures, which leads to particle aggregation and a decrease in specific surface area [14,15]. If it is possible to prepare nanoparticles of transition metal carbides, we can expect the improvement of catalytic activities due to the electronic structures modified by quantum size effects [16,17,18] and isomeric surface atomic arrangement [19,20] as well as the increased surface area of catalysts.

Metal–organic frameworks (MOFs) have gathered attention for catalyst applications [21,22,23]. Also, MOF-derived carbon is synthesized by pyrolysis of MOFs and is now becoming an important group of catalytic materials with metal nanoparticles embedded in a carbon matrix [24,25]. The authors have reported synthesizing single-nanometer-scale copper particles on carbon supports by high pressure and high temperature (HPHT) treatment of MOFs [26,27]. However, MOFs containing only tungsten as a metal element are rarely found in the literature.

High-pressure synthesis has been used to explore new materials because it can provide compounds that are difficult to synthesize at ambient conditions [28,29,30], including catalysts [31,32,33,34,35,36,37]. Under high pressures, the lattice diffusion of atoms is inhibited due to the increased activation energy of diffusion [38,39]. This feature prevents the aggregation of metal atoms during heat treatment, which is beneficial for synthesizing metal carbide nanocomposites. Searching for appropriate precursors and conditions to improve the catalytic activity remains to be explored.

In this paper, we employed organotungsten compounds to synthesize nanostructure-controlled tungsten carbide catalysts using a high-pressure, high-temperature (HPHT) treatment. Since carbon-capsulated iron carbide can be synthesized by pyrolysis of ferrocene [40], it is expected that carbon-supported heterogeneous catalysts consisting of tungsten carbide can be synthesized by pyrolysis of organotungsten compounds. The analysis of the products is of interest since the tungsten carbides have several different species (WC, W_2_C, and *β*-WC_1−x_), and their activity as catalyst support is different [7,8,9]. We report on the structural analysis of tungsten carbides in the HPHT products and their electrocatalytic performance for oxygen-related reactions in alkaline media.

## 2. Materials and Methods

**Figure 1:**
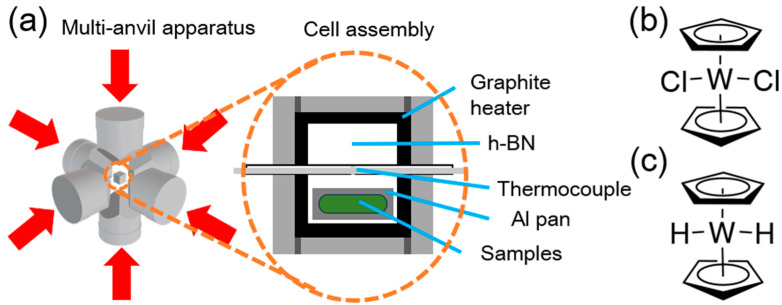
The schematics of (**a**) the cell assembly for HPHT experiments and molecules (**b**) Cp_2_WCl_2_ and (**c**) Cp_2_WH_2_.

Samples were synthesized from pellets of organotungsten compounds by high-temperature, high-pressure processing using a DIA-type cubic anvil high-pressure apparatus (CT-factory, Tokyo, Japan). The schematics of HPHT treatment are illustrated in Figure 1a. The precursors were Bis(cyclopentadienyl)tungsten (IV) dichloride (Cp_2_WCl_2_, Figure 1b) and Bis(cyclopentadienyl) tungsten (IV) dihydride (Cp_2_WH_2_, Figure 1c), purchased from FUJIFILM Wako Chemicals (Tokyo, Japan). The powder of each molecule was crushed and solidified in a vise in an Ar atmosphere in a glove box. It was enclosed in an aluminum pan with an outer diameter of 6 mm and taken out into the air to put it in the cell assembly. It was compressed to the target pressure (4.5 GPa) at room temperature, and the sample temperature, measured by a thermocouple within the cell assembly, was increased from room temperature to the target temperature in about 10 min and was kept for 20 min. The heating was then quenched to room temperature by turning off the power to the heating system and returning to ambient pressure. The obtained samples were finely crushed under ambient conditions using a pestle and used for characterization and electrochemical measurements.

Powder X-ray diffraction (XRD) was measured using MiniFlex-600 (Rigaku, Cu Kα (λ = 1.5405 Å)). Transmission electron microscopy (TEM) and scanning TEM (STEM) observations were performed using a JEM-2010 (JEOL, Akishima, Japan) and Titan G2 60-300 (FEI, Hillsboro, USA). X-ray photoelectron spectroscopy (XPS) was measured using a JPS-9200 (JEOL, Akishima, Japan, using Mg Kα X-rays). Raman spectra were obtained using a Renishaw Invia (Ranishaw, Wotton-under-Edge, UK) with 532 nm laser excitation. The specific surface area of the samples was evaluated by N_2_-sorption measurements. (Belsorp, Microtrac-BEL,Osaka, Japan).

Electrochemical measurements were performed in a 0.1 M KOH solution at room temperature using a rotating disk electrode system (BAS, RRDE-3A, Tokyo, Japan) and potentiostat (Meiden Hokuto, HSV-110, Tokyo, Japan). The working electrode (WE), reference electrode (RE), and counter electrode (CE) for this study were a glassy carbon (GC) rotating disk electrode (0.4 cm diameter), a Hg/HgO reference electrode, and a Pt coil electrode, respectively.

Catalyst inks were prepared as follows: Sample powder (4 mg) was added to a mixture of 60 μL of Nafion 5 wt% solution (Sigma-Aldrich, Burlington, NJ, USA) and 540 μL of 99.5% ethanol (Japan Alcohol Trading, Tokyo, Japan), followed by sonication for 30 min to obtain a uniform ink. The prepared ink (6 μL) was dropped onto a GC disc WE and dried for 30 min. The potential relative to the reversible hydrogen electrode (RHE) was calculated using the following equation: [41].

*E*_RHE_ = *E* _Hg⁄HgO_ + 0.098 + 0.059 × (pH of the electrolyte) (in Volt),

where *E*_RHE_ and *E*_Hg/HgO_ are the potentials relative to the RHE and Hg/HgO reference electrodes, respectively.

## 3. Results and Discussion

**Figure 2:**
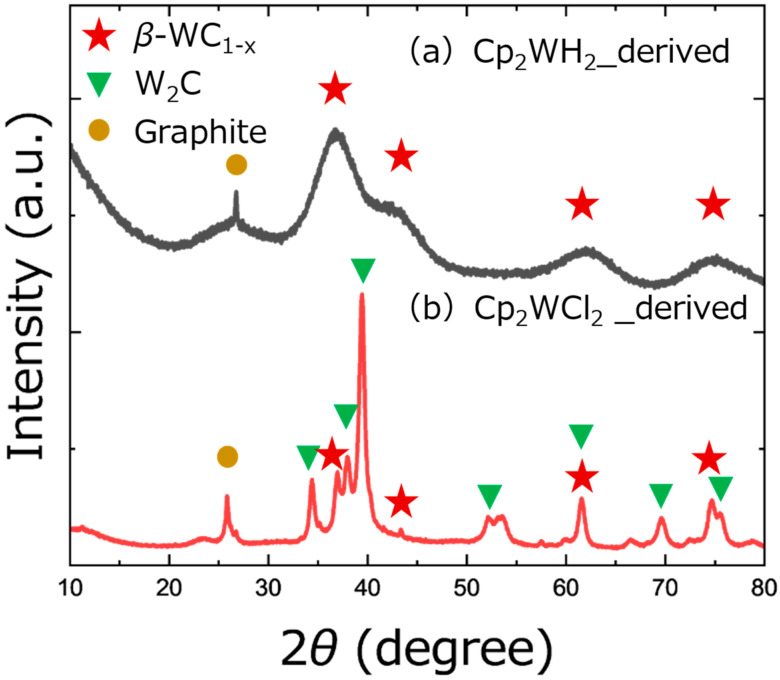
Powder XRD patterns of HPHT-processed samples. (**a**) Cp_2_WH_2_-derived samples. (**b**) Cp_2_WCl_2_-derived samples.

Figure 2 shows the XRD patterns of the Cp_2_WH_2_-derived and Cp_2_WCl_2_-derived samples pyrolyzed under HPHT conditions (4.5 GPa, 600 °C). For both samples, a diffraction peak attributed to graphite was observed at 26.5°, and no diffraction peak corresponding to the tungsten oxides was observed. Diffraction peaks at 36.9°, 42.8°, 62.2°, and 74.6° attributed to *β*-WC_1−x_ [42,43] were observed in both samples, with the Cp_2_WH_2_-derived sample having a broader peak, suggesting that these crystal particles are smaller than those in the Cp_2_WCl_2_-derived sample. Diffraction peaks attributed to W_2_C [42,43] were observed at 34.4°, 38°, 39.5°, 52.2°, 61.7°, 69.6°, and 75.8° only in the Cp_2_WCl_2_-derived sample. Diffraction peaks corresponding to hexagonal WC were not observed. In addition, no tungsten chlorides were formed in the Cp_2_WCl_2_-derived sample. The *β*-WC_1−x_:W_2_C ratio in the Cp_2_WCl_2_-derived sample was analyzed from the simulated diffraction intensity, which resulted in 0.51:1 [44]. It was impossible to analyze the Cp_2_WH_2_-derived sample similarly because the peaks are too broad, but the main component is surely *β*-WC_1−x_. The mechanism of formation of *β*-WC_1−x_ is due to the quenching of the sample, as reported in Refs. [43,44]. We quenched the sample by shutting down the heater immediately after the designated heating period in this experiment.

**Figure 3:**
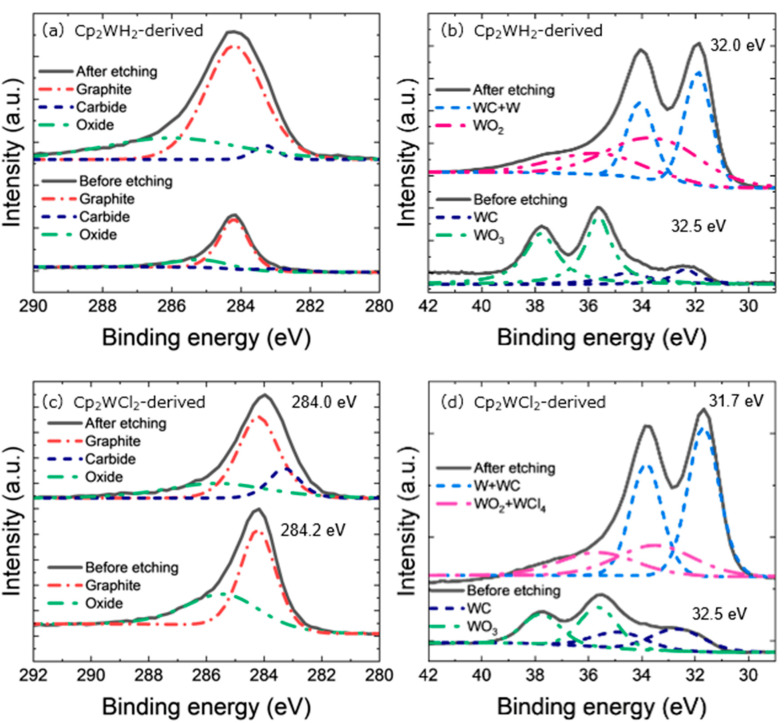
(**a**) C 1s XPS spectra and curve fitting of a Cp_2_WH_2_-derived sample. (**b**) W 4f XPS spectra and curve fitting of a Cp_2_WH_2_-derived sample. (**c**) C 1s XPS spectra and curve fitting of a Cp_2_WCl_2_-derived sample. (**d**) W 4f XPS spectra and curve fitting of a Cp_2_WCl_2_-derived sample. The red line corresponds to graphite, the dark blue line to carbides, the light blue line to carbides and elemental tungsten, the green and dark pink lines to oxides, and the light pink line to oxides and chlorides.

Figure 3 shows the C 1s and W 4f XPS spectra of the Cp_2_WH_2_- and Cp_2_WCl_2_-derived samples. In C 1s of both samples, the peak at 284.2 eV corresponding to graphite was the strongest (Figure 3a,c). The 283 eV signal corresponding to carbide did not appear in the Cp_2_WCl_2_-derived sample before the Ar^+^ etching of the sample surface. Still, it was observed after the etching (estimated removal of 180–400 nm thickness). The etching also shifted the main peak of C 1s to the lower energy side by about 0.2 eV. On the other hand, the Cp_2_WH_2_-derived sample showed a slight signal corresponding to carbides before and after etching. In contrast, both samples showed similar W 4f spectra, with a stronger signal at around 32 eV corresponding to carbides after the etching (Figure 3b,d). These results suggest that tungsten carbide was synthesized, but the sample surface was oxidized. We consider it to be due to the exposure of the sample to air after HPHT treatment (typically 3 h). The role of oxygen species on the ORR catalyst surface is important [45], and we note the existence of adsorped oxygens on the tungsten carbide surfaces, which can be identified as WO_2_.

**Figure 4:**
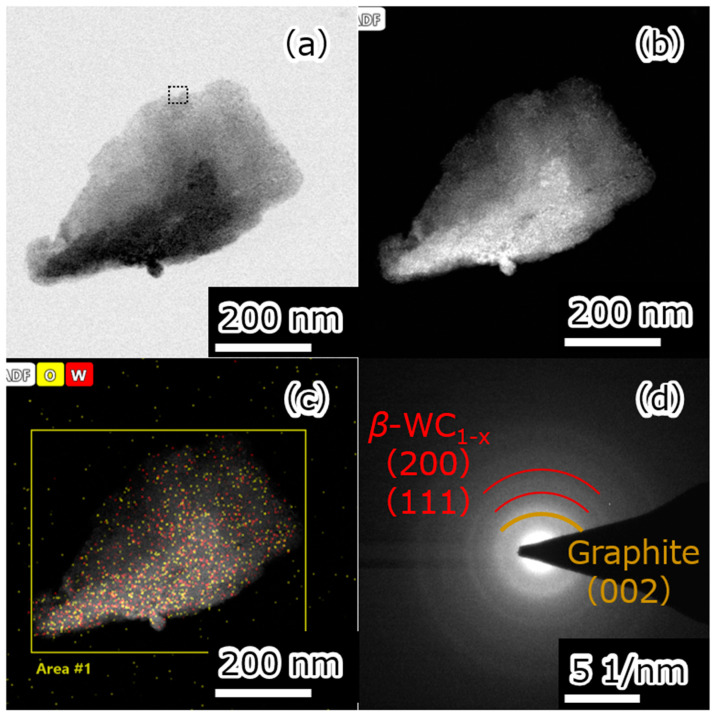
The images of (**a**) STEM, (**b**) HAADF, (**c**) EDS mapping of C and W, and (**d**) electron diffraction pattern in a Cp_2_WH_2_-derived sample particle. The square area enclosed by the dotted line corresponds to Figure 5.

The samples were investigated by STEM. A typical particle in the Cp_2_WH_2_-derived sample is shown in Figure 4. HAADF contrast (Figure 4b) and EDS (Figure 4c) analysis of the particles identified the particle consists of W and C. The result of the quantification in the rectangular region of Figure 4c is shown in Table 1. Comparing the atomic fractions, the ratio of tungsten to carbon was 1:12 after the HPHT synthesis, which is close to the ratio in the precursor (1:10). Electron diffraction analysis was performed on these particles, and diffraction images (Figure 4d) corresponding to *β*-WC_1−x_ and graphite were obtained. This suggests that the particles shown in Figure 4 contain *β*-WC_1−x_ and graphite. This is consistent with the XRD results.

**Figure 5:**
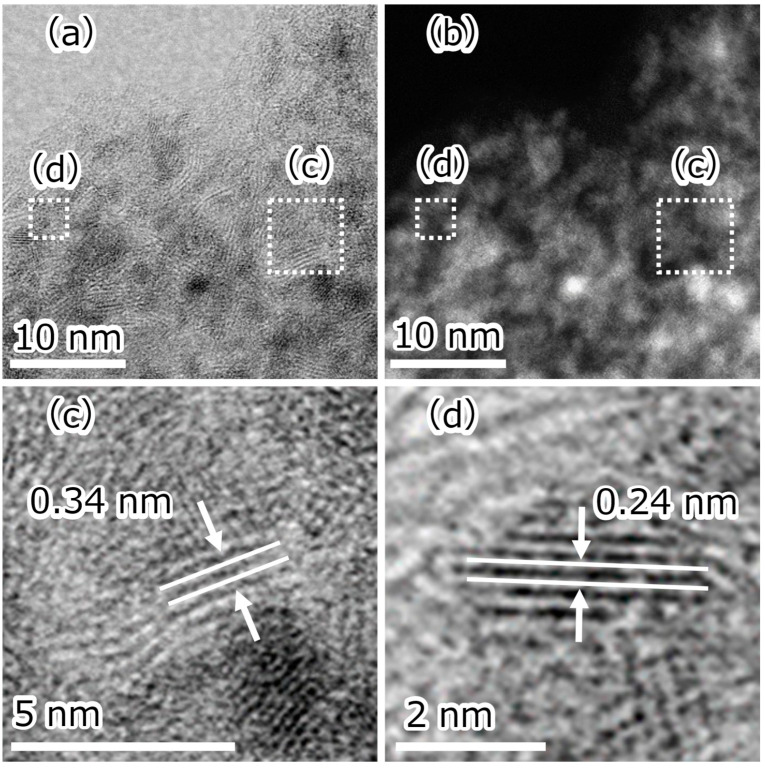
(**a**) STEM image. (**b**) HAADF image. (**c**,**d**) STEM images at high magnification of particles identified as (**c**) graphite and (**d**) *β*-WC_1−x_.

The area marked by a square in Figure 4a was further magnified (Figure 5). Several single nanoscale stripes were shown, and the HAADF-STEM contrast revealed both carbon- and W-derived particles (Figure 5a,b). A magnified view of the carbon-derived particles is shown in Figure 5c, showing a fringe spacing of about 3.4 Å, corresponding to 3.42 Å on the (0 0 2) lattice plane of graphite. A magnified view of the tungsten-derived particles is shown in Figure 5d, which shows a fringe spacing of about 2.4 Å, corresponding to a (1 1 1) lattice plane of 2.43 Å for *β*-WC_1−x_. The size of *β*-WC_1−x_ was about 2 nm. These results are consistent with XRD, electron diffraction patterns, and EDS results.

**Figure 6:**
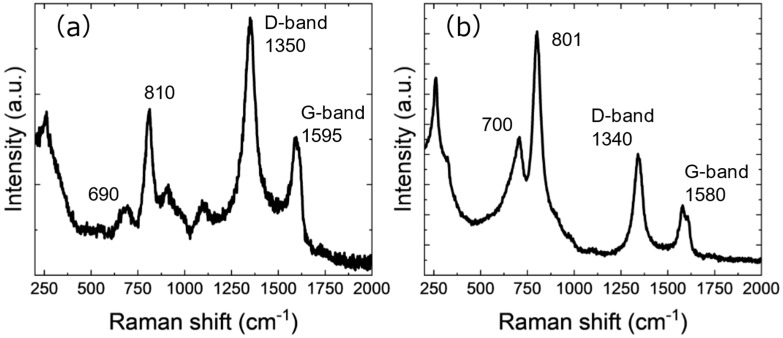
Raman spectra of (**a**) Cp_2_WH_2_-derived and (**b**) Cp_2_WCl_2_-derived samples excited by 532 nm laser.

Raman spectra are shown in Figure 6. Broad peaks around 1340–50 cm^−1^ and 1580–95 cm^−1^ correspond to D and G bands of carbon [46]. In the case of bulk crystalline graphite, the peaks are sharper, and the intensity of the D-band peak is smaller. In the case of Cp_2_WH_2_-derived samples, the STEM results show the synthesis of nano-sized graphite, and this small crystallite size is considered to be responsible for the observed spectra. The presence of graphite in the Cp_2_WCl_2_-derived sample is also consistent with the XRD and electron diffraction images. In addition, bands associated with the W-C stretching mode [43,46] are located at about 801 and 810 cm^−1^ for the Cp_2_WCl_2_- and Cp_2_WH_2_-derived samples, respectively. In addition, bands attributed to the initial oxidation of WC (WC-O)/WC [47,48] are located at about 700 and 690 cm^−1^, respectively. The Raman spectra thus confirm the synthesis of tungsten carbides.

**Figure 7:**
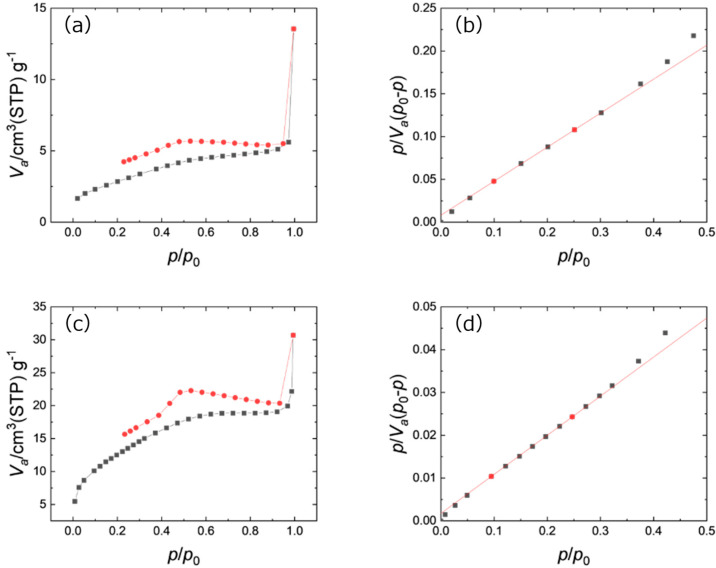
(**a**) Nitrogen adsorption(black)–desorption(red) isotherms and (**b**) BET plots of Cp_2_WH_2_-derived samples. Squares (black and red) are data from the experiment and the red line is the line intersecting two points indicated in red. (**c**) Nitrogen adsorption–desorption isotherms and (**d**) BET plots of Cp_2_WCl_2_-derived samples. The meaning of colors and shapes are the same as (**b**).

The specific surface areas of the samples were evaluated by using N_2_ adsorption–desorption isotherms (Figure 7a,c). Both isotherms have similar hysteresis and show a type II isotherm characteristic of materials with no pores or macro-sized pores. Brunauer–Emmett–Teller (BET) plots were made based on these isotherms (Figure 7b,d). The specific surface area calculated from the BET plot was 10.8 m^2^ g^−1^ and 46.8 m^2^ g^−1^ for the Cp_2_WH_2_-derived and the Cp_2_WCl_2_-derived samples, respectively.

**Figure 8:**
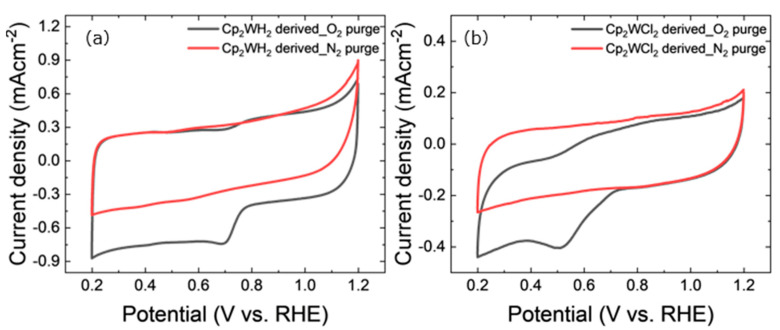
CV curves of (**a**) Cp_2_WH_2_-derived samples and (**b**) Cp_2_WCl_2_-derived samples at 10 mVs**^−^**^1^. The red and black curves are measured in N_2_-saturated and O_2_-saturated 0.1 M KOH aqueous solutions, respectively.

To investigate the electrocatalytic activity against ORR, cyclic voltammetry (CV) curves were measured in N_2_-saturated and O_2_-saturated 0.1 M KOH solutions, as shown in Figure 8. Both samples exhibited cathodic peaks in the range of 0.5–0.8 V vs. RHE only in the O_2_-saturated solution. This means that the reaction attributed to the cathodic peak is an O_2_-related reduction reaction.

**Figure 9:**
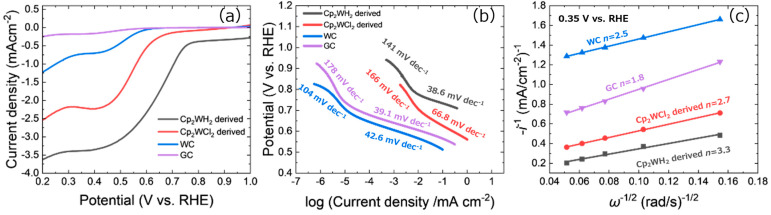
(**a**) LSV curves of the ORR for the products in O_2_-saturated 0.1 M KOH aqueous solution at rotation rates of 1600 rpm and scan rates of 10 mV s^−1^. (**b**) Tafel plots of the ORR for the products. The constant current was subtracted. (**c**) Koutecký–Levich plots of the products at 0.35 V vs. RHE, where *i* and *ω* are disk current density and disk rotating speed, respectively.

Figure 9a shows linear sweep voltammetry (LSV) results measured using a rotating disk electrode (RDE) in an O_2_-saturated 0.1 M KOH aqueous solution, where the ORR activity was evaluated in detail. For comparison, commercial WC bulk and glassy carbon (GC) were measured without ink in addition to the HPHT-synthesized samples. The starting potentials of the Cp_2_WH_2_-derived samples, Cp_2_WCl_2_-derived samples, WC, and GC were 0.75, 0.67, 0.60, and 0.58 V vs. RHE, respectively. The synthesized samples performed better than the commercial hexagonal WC powder, and the Cp_2_WH_2_-derived sample showed the best performance. Since the XRD and STEM results showed a smaller crystallite size (2 nm) of tungsten carbide and dominance of the *β*-WC_1−x_, the electronic structure of nano-sized *β*-WC_1−x_ is responsible for the high activity. It is noted that Pt nanoparticles supported by *β*-WC_1−x_ showed better catalytic activity over those supported by other tungsten carbides [49], suggesting the importance of the electronic structure of the material. Tafel plots were generated from the LSV curves in Figure 9a to evaluate the reaction kinetics of ORR in detail (Figure 9b). The Tafel slope of the Cp_2_WH_2_-derived sample showed the steepest slope, meaning it has the most significant efficiency.

Electron transfer numbers in ORR were analyzed using Koutecký–Levich plots. As shown in Figure 9c, each plot drew a good straight line, and the reaction electron numbers *n* were calculated from the slope of each plot. The *n* values of the Cp_2_WH_2_-derived samples, Cp_2_WCl_2_-derived samples, WC, and GC, were 3.3, 2.7, 2.5, and 1.8, respectively. If the number of electrons is close to four, it indicates that the reaction proceeds mainly to produce H_2_O; if it is close to two, it suggests that the reaction proceeds primarily to produce hydrogen peroxide. Among these, the Cp_2_WH_2_-derived sample is closest to the reaction that produces H_2_O. The Cp_2_WH_2_-derived sample is the best catalyst among the synthesized samples because it has the largest Tafel gradient, the largest onset potential, and the reaction electron number closest to the widely applicable H_2_O-producing reaction.

**Figure 10:**
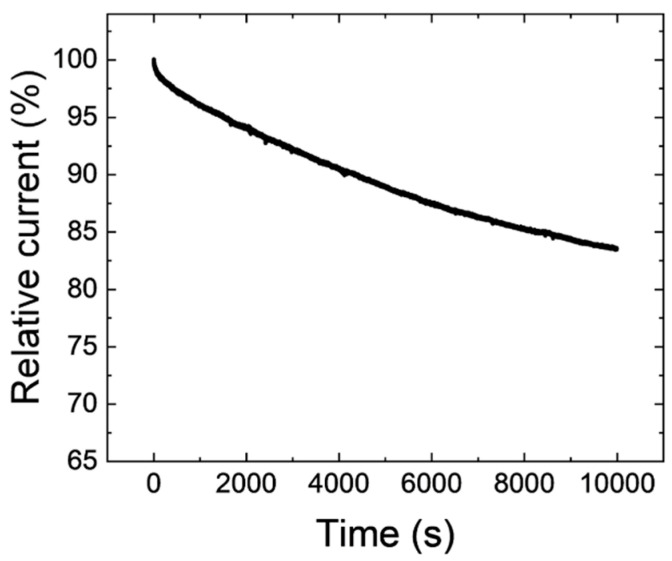
The chronoamperometric curves of Cp_2_WH_2_-derived samples in O_2_-saturated 0.1 M KOH electrolyte at 0.60 V and 1600 rpm. The first 10 **s** showing a large decrease were omitted as initial degradation.

Figure 10 shows the stability of the Cp_2_WH_2_-derived catalyst from the chronoamperometric measurement in O_2_-saturated 0.1 M KOH electrolyte at 0.60 V and 1600 rpm. It shows 83% performance after a 10,000 s operation, and the decrease rate slows down as the operation continues. Although it is necessary to improve lifetime before using this catalyst in practice, the lifetime performance is promising, considering the brittle nano-sized composite structure.

As shown above, our Cp_2_WH_2_-derived *β*-WC_1−x_ nanoparticles showed the ORR onset potential of 0.75 V vs. RHE, which is 0.17 V less than that of Pt. We compare this performance with the tungsten carbide (WC_x_) catalysts synthesized by various methods. WC_x_ prepared from solid state reaction [50], from combustion [51], and from pyrolyzing W-adsorbed polymer [52] showed 0.1–0.2 V inferior onset for ORR compared with the present results. However, hexagonal WC nanoparticles combined with nitrogen-doped carbon aerogel performed even better than Pt/C [53]. This can be compared with other co-catalysis systems with WC_x_ studied to reduce the use of precious metals such as sub-monolayer Pt [49,54] and other metals or inorganic compounds, some of which showed good performance comparable to Pt/C.

**Figure 11:**
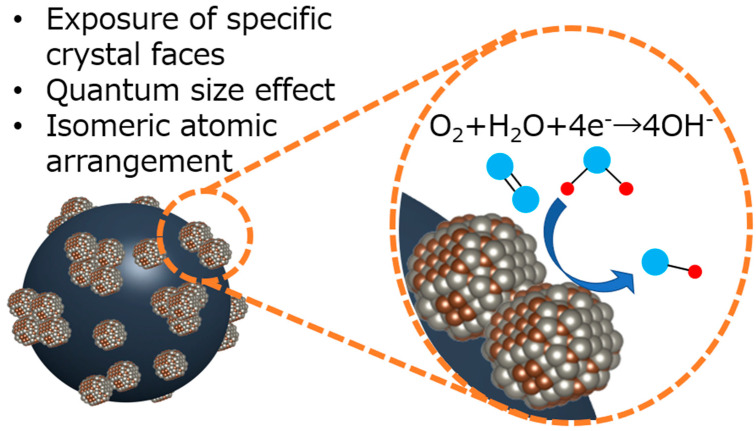
Possible mechanisms for improved catalytic performance of tungsten carbide nanoparticles.

It is difficult to discuss the detailed mechanism at this stage, but the exposure of specific crystal faces, novel atom arrangements, and electronic structure change from the quantum size effect are the candidates that are actively examined theoretically [55]. This is illustrated in Figure 11.

## 4. Conclusions

We synthesized tungsten carbide electrocatalysts by high-pressure and high-temperature treatment of organotungsten compounds: the Cp_2_WH_2_-derived sample contained only *β*-WC_1−x_, while the Cp_2_WCl_2_-derived sample contained both W_2_C and *β*-WC_1−x_ carbides. STEM-HAADF analysis revealed that the Cp_2_WH_2_-derived sample was a composite from single nanoscale (~2 nm) graphite and *β*-WC_1−x_ crystals. These single nanoscale crystals may have contributed to the better catalytic performance of the Cp_2_WH_2_-derived sample in the ORR than that of the Cp_2_WCl_2_-derived sample and bulk WC. We conclude that HPHT processing of organometallic compounds can be a new approach to synthesizing metal carbide catalysts [56].

## Figures and Tables

**Table 1:** EDS quantitation results.

Element	Atomic Fraction/%
W	5.57
C	68.1
O	21.5
Cu (from Cu TEM grid)	4.84

## Data Availability

Data can be obtained from the contact author on request.
